# A Panel of Bile Volatile Organic Compounds Servers as a Potential Diagnostic Biomarker for Gallbladder Cancer

**DOI:** 10.3389/fonc.2022.858639

**Published:** 2022-03-30

**Authors:** Xin Zhang, Xinru Gui, Yanli Zhang, Qi Liu, Liqiang Zhao, Jingxian Gao, Jian Ji, Yi Zhang

**Affiliations:** ^1^Department of Clinical Laboratory, Qilu Hospital of Shandong University, Shandong University, Jinan, China; ^2^Department of Clinical Laboratory, Shandong Provincial Third Hospital, Jinan, China; ^3^Department of Research and Development, Hanon Advanced Technology Group Co., Ltd, Jinan, China

**Keywords:** gallbladder cancer, volatile organic compounds, diagnosis, biomarker, bile

## Abstract

As no reliable diagnostic methods are available, gallbladder cancer (GBC) is often diagnosed until advanced stages, resulting in a poor prognosis. In the present study, we assessed whether volatile organic compounds (VOCs) could be used as a diagnostic tool for GBC. The VOCs in bile samples collected from 32 GBC patients were detected by gas chromatography-ion mobility spectrometry (GC-IMS), and 54 patients with benign gallbladder diseases (BGD) were used as controls. Both principal component analysis and unsupervised hierarchical clustering analysis gave a clear separation of GBC and BGD based on the bile VOC data collected from GC-IMS. A total of 12 differentially expressed VOCs were identified, including four upregulated (cyclohexanone, 2-ethyl-1-hexanol, acetophenone, and methyl benzoate) and eight downregulated [methyl acetate, (E)-hept-2-enal, hexanal, (E)-2-hexenal, (E)-2-pentenal, pentan-1-ol, 1-octen-3-one, and (E)-2-octenal] in GBC compared with BGD. ROC analysis demonstrated a 12-VOC panel con-structed by four machine learning algorithms, which was superior to the traditional tumor marker, CA19-9. Among them, support vector machines and linear discriminant analysis provided the highest AUCs of 0.972, with a sensitivity of 100% and a specificity of 94.4% in the diagnosis of GBC. Collectively, VOCs might be used as a potential tool for the diagnosis of GBC.

## Introduction

Gallbladder cancer (GBC) is one of the most common malignant tumors of the biliary system in Eastern Asia with high mortality (1). In 2020, there are an estimated 115,949 new cases and 84,695 GBC-related deaths worldwide (2). At present, surgical resection is the most effective treatment for GBC (3). Unfortunately, due to the atypical clinical symptoms, the patients often are diagnosed at advanced stages and can not accept surgical treatment, resulting in a poor prognosis for this aggressive disease. Detection in an earlier stage of GBC and removal of precancerous lesions will reduce the disease burden and mortality rate. However, currently used tumor biomarkers, such as CA 19-9 and CA 242, have low sensitivity and specificity (4–6). Moreover, they have nonspecific elevations in benign gallbladder diseases (BGD), such as gallstones, cholecystitis, polyps, and gallbladder adenomyosis (7, 8). Therefore, it is urgently necessary to identify new biomarkers for the early clinical diagnosis.

Metabolomics is one of the most promising approaches for identifying biomarkers of disease and increasing understanding of metabolic processes in cancer (9). As an important part of metabolism products, volatile organic compounds (VOCs) reflect the metabolic changes produced in a variety of different biochemical reactions in the human body. VOCs are a type of organic matter that exists in the form of steam at room temperature, which can be divided into aromatic hydrocarbons, alkanes, olefins, halohydrocarbons, esters, and ketones. Due to their distinct odors, VOCs emitted from different substrates can be detected by gas chromatography-mass spectrometry (GC-MS), gas chromatography-ion mobility spectrometry (GC-IMS), electronic nose (E-Nose), or even trained sniffer dogs (10–12). Bhatt et al. (13) have studied the plasma metabolomics of 20 patients with esophageal adenocarcinoma and 19 patients with gastroesophageal reflux disease, disclosing nine VOCs and unveiling significant differences between the two groups. Lima et al. (14) have performed the GC-MS to detect the volatile metabolomic signature of urine and established a panel of six volatile biomarkers for the identification of prostate cancer. When compared with other fecal-based techniques, VOCs emitted from feces, such as propan-2-ol, hexan-2-one, and ethyl 3-methyl-butanoate, have a superior diagnostic capability for the diagnosis of colorectal cancer (15, 16). Until now, little is known about VOCs in GBC, and their potential utility to serve as biomarkers for GBC diagnosis remains largely unclear.

In the present study, we performed GC-IMS to obtain the metabolomic profiling of VOCs in bile from patients with GBC and BGD. Moreover, we aimed to develop a volatile biomarker panel that could act as a minimally invasive approach for the early detection of GBC. To the best of our knowledge, we, for the first time, showed that VOCs could be used as bile biomarkers for the diagnosis of GBC.

## Materials and Methods

### Study Population

In the present study, patients who were older than 18 years and histologically diagnosed with GBC were recruited from 2018 to 2021 in Qilu Hospital of Shandong University. Inclusion criteria were set as follows: 1) patients without any history of other malignant tumor or anti-cancer therapy, 2) patients who were cooperative with supplying fresh bile sample and complete medical records, and 3) patients who underwent radical resection and reported GBC by pathological examination. Patients with BGD, such as cholecystitis and gallbladder polyps, who met the above-mentioned conditions except for the pathologically reported GBC, were included as controls. The experimental scheme was approved by the Ethics Committee of Qilu Hospital of Shandong University, and the informed consent were got from each patient.

### Sample Preparation

Bile samples were collected when the patient was first treated with endoscopic retrograde cholangiopancreatography (ERCP) or percutaneous transhepatic cholangiodrainage (PTCD). The collected specimens were centrifuged at 3,000 rpm for 10 min at 4°C. The supernatants were aliquoted and stored at -80°C immediately.

### Analysis of the VOCs in Bile

VOC profiles from bile samples were detected using GC-IMS (G.A.S. Dortmund, Germany). All samples underwent the same procedure. Briefly, 0.5 mL bile was placed in each headspace bottle and incubated at 80°C for 10 min. Subsequently, 1 mL headspace gas was extracted for analysis. Nitrogen was used as the carrier air. The IMS drift gas was always maintained at 150 mL/min, while the initial flow rate of the carrier air was maintained at 2 mL/min for 2 min, and then it was linearly increased to 150 mL/min in 10 min. Other major experimental parameters were as follows: drift tube temperature: 45°C; gas chromatography column temperature: 60°C; inlet-chromatography column converter temperature: 60°C; column-migration tube converter temperature: 45°C; ion mode: positive ion mode. Each analysis was conducted in triplicate.

### Statistical Analysis

The software R (x64 3.6.2) and the software package “ggord” were used for principal component analysis (PCA). The level of each VOC was compared with Mann–Whitney U test. The area under the curve (AUC) was calculated on the receiver operating characteristic (ROC) curve and compared using MedCalc 9.3.9.0. The analysis of machine learning was carried out using Matlab R2016a (Python Software Foundation, Beaverton, USA) based on the Statistics and Machine Learning Toolbox. Based on identified VOCs, decision tree (DT), support vector machines (SVM), linear discriminant analysis (LDA), gradient enhancement machines (GBMs), and K-nearest neighbor (KNN) were used for classification. Hierarchical 10-fold cross-validation was used to optimize the parameters of the training cohort.

## Results

### Clinical Characteristics of GBC and BGD Patients

A cohort consisting of 86 patients with definite pathological diagnoses, including 32 GBC patients (age 52–77 years, mean 63) and 54 BGD patients (age 56–66 years, mean 59), were included in the present study. Moreover, 70% recruited subjects were randomly selected as a training cohort (n = 24 GBC and n = 36 BGD), while the remaining 26 samples (n = 8 GBC and n = 18 BGD) were set as a test cohort. There were no significant differences between the two cohorts in terms of age, sex, and some biochemical indexes. **Table 1** lists more detailed clinical characteristics of these patients.

**Table 1:** Clinical characteristics of GBC and BGD patients.

Charateristics	GBC		BGD	
	Training set	Test set	Training set	Test set
Cases	24	8	36	18
age (years)^*^	60.6 ± 10.3	58.9 ± 6.5	62.1 ± 10.3	57.4 ± 16.4
Male/Female	16/8	5/3	23/13	10/8
ALB (g/L)^#^	37.3 (32.7-39.9)	41.0 (35.3-43.2)	42.0 (40.3-45.4)	41.4 (37.8-43.5)
AKP (U/L)^#^	337.5 (140.3-469.8)	330.0 (261.5-505.3)	119.0 (66.0-131.0)	151.5 (81.3-368.5)
AST (U/L)^#^	64.0 (32.5-155.8)	85.5 (43.3-121.5)	37.5 (21.0-45.0)	42.0 (17.3-81.0)
ALT (U/L)^#^	92 (45.5-184.0)	91.5 (68.0-202.3)	38.5 (13.0-74.0)	50.5 (16.5-100.5)

*Data represents mean ± standard deviation; ^#^Data represents the median (interquartile range). GBC, gallbladder cancer; BGD, benign gallbladder diseases; ALB, albumin; AKP, alkaline phosphatase; AST, aspartate aminotransferase; ALT, alanine aminotransferase.

### Coefficient of Variation for VOC Analysis With GC-IMS

**Figure 1:**
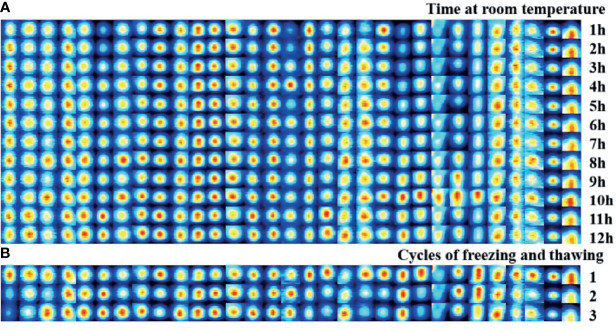
Stability evaluation of bile VOCs analysis with GC-IMS. **(A)** Room-temperature stability was assessed in the same samples that measured in parallel every hour within 12 hours, with a total of 12 injections. **(B)** Freeze/thaw sta-bility evaluation was assessed in the same samples that measured within three freeze-thaw cycles.

Room-temperature stability was assessed in the same samples that were measured in parallel every hour within 12 h, with a total of 12 injections. **Figure 1A** shows that the intensity difference of the selected signal peaks in the 12 repeated determinations was little under the same experimental conditions. The average CV for the bile at room temperature for 12 h was within 10% (**Supplementary Table 1**). **Figure 1B** presents that repeated freeze-thaw cycles might affect the VOC composition, while the average CV of VOCs within three freeze-thaw cycles was less than 10% (**Supplementary Table 2**). In the present study, each sample was detected within 3 h after being thawed at room temperature.

### VOC Profile Analysis in GBC and BGD Patients

**Figure 2:**
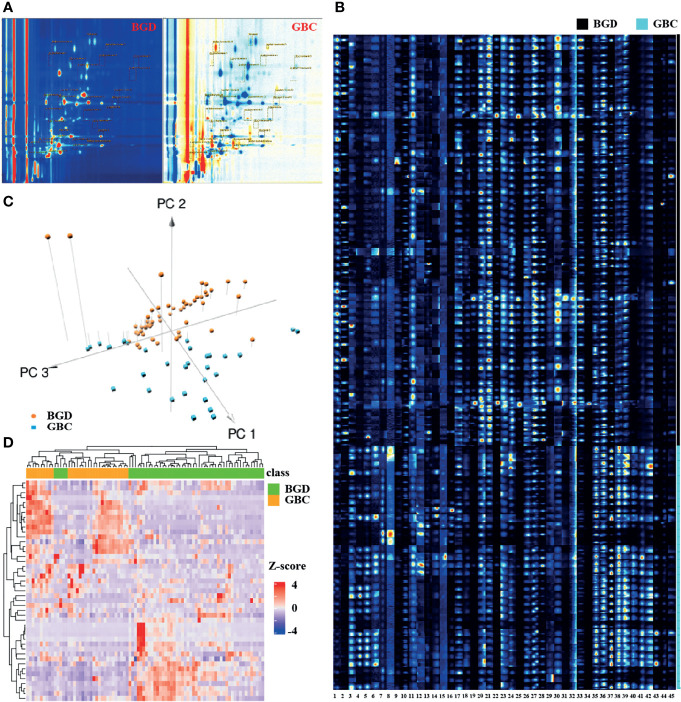
Bile VOCs profile analysis. **(A)** 2D spectral map for BGD and GBC, and each point represent a signal peak. **(B)** GC-IMS signals detected in the bile of BGD and GBC patients. **(C)** A 3-dimensional scatterplot generated from PCA for of VOCs profiles in BGD and GBC patients. **(D)** Heatmap of GC-IMS VOCs profiles in BGD and GBC patients. Note: 1. Unknown-1, 2. Unknown-2, 3. Unknown-3, 4. Unknown-4, 5. Unknown-5, 6. Unknown-6, 7. Methyl benzoate-M, 8. Methyl benzoate-D, 9. pentan-1-o1, 10. 2-heptanone, 11. Nonanal, 12. methyl acetate-M, 13. methyl acetate-D, 14. Methyl isobutyl ketone, 15. 1-propene-3-methylthio, 16. 2-Pentanone, 17. Butyl acetate, 18. 1-octen-3-one-M, 19. 1-octen-3-one-D, 20. (E)-hept-2-enal-1, 21. (E)-hept-2-enal-2, 22. (E)-hept-2-enal-3, 23. (E)-2-octenal-1, 24. (E)-2-octenal-2, 25. (E)-2-octenal-3, 26. Hexanal-1, 27. Hexanal-2, 28. Hexanal-3, 29. (E)-2-pentenal-1, 30. (E)-2-pentenal-2, 31. (E)-2-pentenal-3, 32. (E)-2-hexenal-1, 33. (E)-2-hexenal-2, 34. (E)-2-hexenal-3, 35. cyclohexanone-1, 36. cyclohexanone-2, 37. cyclohexanone-3, 38. 2-ethyl-1-hexanol-1, 39. 2-ethyl-1-hexanol-2, 40. 2-ethyl-1-hexanol-3, 41 Acetophenone-1, 42. Acetophenone-2, 43. Acetophenone-3, 44. Benzaldehy de-M, 45. Benzaldehy de-D.

The VOC was characterized by the molecular gas chromatography preservation index, and the migration time of molecular ions was measured and quantified according to the signal peak strength. For each sample, we would generate the 3D data (retention index, migration time, and peak strength) (**Supplementary Figure S1**). Our VOCs were selected from a 2D spectral map (the vertical view of 3D spectra, with color to indicate peak strength), and each point represented a signal peak (**Figure 2A**). **Figure 2A** shows that we could visually see the difference in the VOC between a GBC sample and a BGD sample, with red representing a higher concentration of the substance in the bile of GBC compared with BGD, and blue representing a lower concentration.

Using VOCal software (v0.1.1) with a GC-IMS library, a total of 45 VOC peaks were manually selected based on retention index and migration time in all patients. These species (peaks) included 19 defined substances and six unknown substances (**Figure 2B**). A 3D scatterplot generated from PCA demonstrated that the VOC profile of GBC patients generally differed from that of BGD, and the respective clustering trend could be observed (**Figure 2C**). Unsupervised hierarchical clustering analysis showed a clear separation of GBC and BGD (**Figure 2D**). These data indicated that VOCs had potential as biomarkers for the diagnosis of GBC.

### Quantitative Analysis of VOCs in the Training Cohort

**Figure 3:**
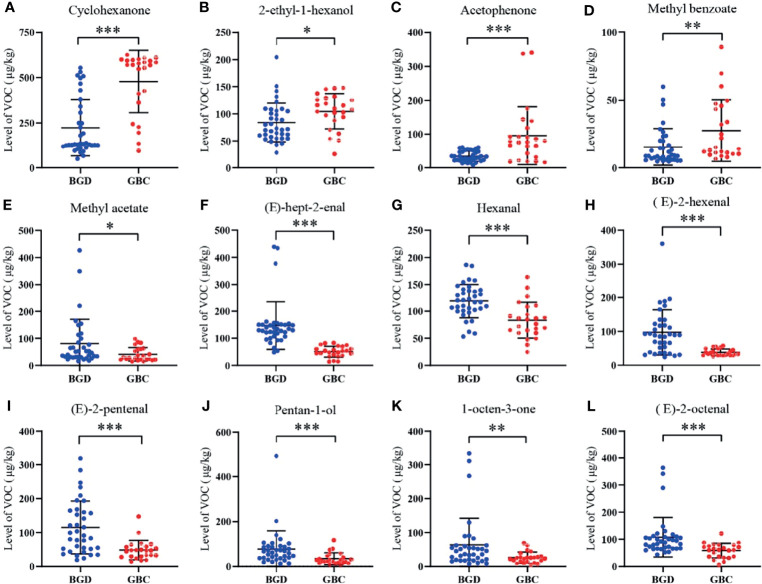
Quantitative analysis of VOCs in the training cohort. The levels of Cyclohexanone **(A)**, 2-ethyl-1-hexanol **(B)**, Acetophenone **(C)**, Methyl benzoate **(D)**, Methyl acetate **(E)**, (E)-hept-2-enal **(F)**, Hexanal **(G)**, (E)-2-hexenal **(H)**, (E)-2-pentenal **(I)**, Pentan-1-ol **(J)**, 1-octen-3-one **(K)**, (E)-2-octenal **(L)**. *P < 0.05, **P < 0.01, ***P < 0.001(Mann–Whitney U test). Data represents the median (interquartile range).

The internal standard method was used for quantification according to the peak volume of VOCs. Briefly, 10 μL 4-methyl-2-pentanol at a concentration of 15 μL/L was used as the internal standard, which was added to each sample. **Figure 3** shows that 12 differentially expressed VOCs were identified, including four up-regulated (cyclohexanone, 2-ethyl-1-hexanol, acetophenone, and methyl benzoate) and eight down-regulated [methyl acetate, (E)-hept-2-enal, Hexanal, (E)-2-hexenal, (E)-2-pentenal, pentan-1-ol, 1-octen-3-one, and (E)-2-octenal] in GBC patients compared with BGD patients. The other seven VOCs showed no significant difference between GBC and BGD (**Supplementary Table 3**).

ROC curve analyses realized that pentan-1-ol, (E)-2-octenal, (E)-hept-2-enal, (E)-2-hexenal, (E)-2-pentenal, cyclohexanone, and acetophenone were robust in distinguishing GBC patients from BGD patients, with AUCs>0.75 (**Table 2**). Among them, (E)-hept-2-enal was significantly superior to CA19-9, a routine clinically used marker in the diagnosis of GBC.

**Table 2:** The AUC, sensitivity and specificity of each VOC for GBC diagnosis.

VOC molecular	AUC (95% CI)	Sensitivity (%)	Specificity (%)	P^*^
Methyl benzoate	0.711 (0.580 - 0.821)	87.5	52.8	0.361
Pentan-1-ol	0.774 (0.648 - 0.872)	70.8	80.6	0.790
Methyl acetate	0.675 (0.542 - 0.791)	54.2	80.6	0.166
1-octen-3-one	0.704 (0.572 - 0.815)	79.2	63.9	0.352
(E)-2-octenal	0.799 (0.675 - 0.891)	95.8	52.8	0.995
2-ethyl-1-hexanol	0.694 (0.562 - 0.807)	75.0	66.7	0.156
(E)-hept-2-enal	0.965 (0.883 - 0.995)	100	88.9	0.011
Hexanal	0.787 (0.662 - 0.882)	75.0	83.3	0.903
(E)-2-hexenal	0.834 (0.715 - 0.917)	100	69.4	0.658
(E)-2-pentenal	0.792 (0.667 - 0.886)	91.7	63.9	0.940
Cyclohexanone	0.874 (0.763 - 0.945)	66.7	97.2	0.165
Acetophenone	0.795 (0.671 - 0.888)	70.8	100	0.658

^*^Compared with CA19-9 using MedCalc 9.3.9.0 software. AUC, area under the curve; CI, confidence interval; VOC, volatile organic compounds; GBC, gallbladder cancer.

### Diagnostic Performance of VOCs With Machine Learning Algorithms

**Figure 4:**
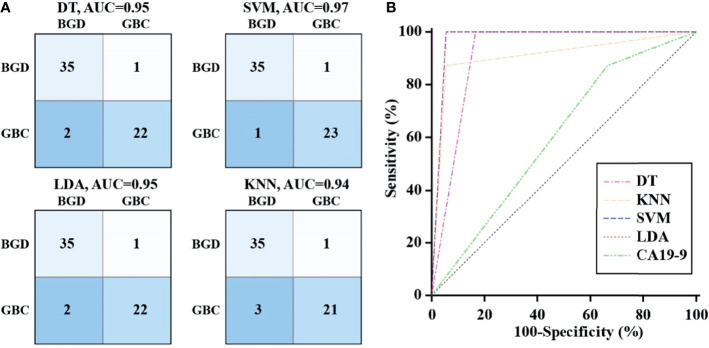
Diagnostic performance of VOCs with machine learning algorithm. **(A)** The confusion matrix of models constructed by DT, SVM, LDA and KNN in the training cohort. **(B)** ROC curves analysis for machine learning models and CA19-9 in differentiating GBC from BGD in the test cohort.

To ensure the accuracy of the diagnostic model under limited data sets, the machine learning method was used to analyze the heterogeneous signal patterns of gallbladder diseases. Coupled with the above-mentioned 12 VOCs, four popular machine learning algorithms (DT, SVM, LDA, and KNN) were used to construct diagnostic models. **Figure 4A** shows the prediction and classification of the model for the training cohort, with each AUC>0.9 in distinguishing GBC patients from BGD patients. In the test cohort, ROC analysis demonstrated that the machine learning models performed better than CA19-9 in differentiating GBC from BGD (**Figure 4B**). Among them, SVM and LDA provided the highest AUCs of 0.972, with a sensitivity of 100% and a specificity of 94.4% (**Supplementary Table 4**).

## Discussion

There are still numerous challenges for us to more accurately diagnose GBC. In the present study, we explored the potential role of GC-IMS in the detection of GBC and thus reported several meaningful findings. First, GC-IMS could discriminate between bile samples collected from patients with GBC and BGD. Second, we identified 12 specific VOCs, which might play a relevant role in assessing GBC. Third, the model based on the VOC profile allowed for accurate discrimination between GBC and BGD groups. However, this observation was limited to a small cohort of patients.

There is increasing interest in the application of VOCs in exhaled breath for diagnosing a variety of cancers (17, 18). Since VOCs exist in the form of steam, a large number of compounds enter into a gaseous state. Moreover, these molecules diffuse into the blood and are carried to the alveolar membrane, where they spread to the lungs and are exhaled during breathing (19). However, VOCs in exhaled breath may be altered by external factors, such as the surrounding environment, diet, and bacteria (20). Bile is aspirated during ERCP, which is less likely to be affected by confounding factors. Therefore, VOCs in bile may better represent the metabolic activities of surrounding cells in the biliary tract. Navaneethan et al. (21) have reported that the measurement of VOCs in bile is useful to distinguish patients with cholangiocarcinoma from primary sclerosing cholangitis. Recently, they have performed another prospective observational study and found that VOCs in the biliary fluid can help accurately discriminate pancreatic cancer from chronic pancreatitis (22). Our study presented for the first time that bile headspace VOCs were significantly altered in GBC patients. Moreover, both PCA plots and unsupervised hierarchical clustering analysis revealed a clear separation for GBC and BGD cases, suggesting that the VOC profile of GBC patients generally differed from that of BGD patients.

Although GC-IMS has been first used in the detection of bile VOCs, it has been successfully implemented feasibly into others. Maxine and her colleagues have observed a significant difference in fecal VOC profiles using GC-IMS between coeliac disease and refractory coeliac disease (23). Based on urinary VOC profiles, both GC-IMS and GC-TOF-MS methods can establish an interdependence among bladder cancer, prostate cancer, and non-cancerous samples (24). A similar study conducted by Daulton et al. has suggested that GC-IMS and GC-TOF-MS can distinguish pancreatic ductal adenocarcinoma from healthy controls, whereas only GC-IMS can accurately discriminate chronic pancreatitis from healthy controls (25). In the present study, we performed a preliminary analysis to assess the use of the GC-IMS in the diagnosis of GBC. GC-IMS possesses a strong separation capability of complex components with the ultra-high sensitivity of the ion migration spectrum to detect trace volatiles of 10^-9^ or less without enrichment and concentration. Meanwhile, the detection time is shortened to 10 min, which significantly improves the simplicity of detection operation, detection time, and efficiency. In contrast, traditional methods usually need to concentrate the samples, and the detection process can last more than 1 h. It was worth noting that the advantage of this study was to explore the experimental conditions by using orthogonal experiments and to test the effects of room temperature and repeated freeze-thaw cycles on VOCs. The sample was relatively stable within 12 h at room temperature, which facilitated the detection.

In this study, 12 specific VOC molecules linked to GBC were identified. Some of them have also been suggested as potential biomarkers in breath or stool for other diseases. For instance, cyclohexanone is associated with colorectal cancer (26), breast cancer (27), and lung cancer (28). 2-Ethyl-1-hexanol is elevated in the detection of VOCs in patients with lung cancer (28), colorectal cancer (29), and prostate cancer (30). The detailed mechanism of VOC production is not well understood until now, while some researchers have pointed out that these compounds may act directly on the enzyme function (31). Aldehyde dehydrogenase is an important catalyst in the human body, resulting in the oxidization of aldehydes to carboxylic acid (32). Moreover, the carboxylic acids further participate in the synthesis of intracellular lipids, providing materials for the cell membrane (33). With the vigorous metabolism of tumor cells, the activity of acetaldehyde dehydrogenase is increased (34, 35). Therefore, this may explain why the levels of (E)-hept-2-enal, (E)-2-hexenal, (E)-2-pentenal, (E)-2-octenal, and hexanal are reduced in the bile of tumor patients. These findings were consistent with some studies that volatile aldehydes are decreased in tumor cells (36, 37).

At present, the diagnosis of GBC mainly depends on the clinical manifestations of the disease, CT, B-ultrasound, and other imaging examinations, while these approaches are too subjective, and there are too many external interference factors. Serum CA19-9 test is one of the few non-invasive markers for clinicians to make a preliminary diagnosis (38, 39). However, it only provides limited sensitivity and poor specificity for GBC diagnosis (40). We found that detection of serum CA19-9 had a sensitivity of 87.5% and a specificity of 33.3% using the given cutoff value, with an AUC of 0.604. Among the identified VOCs, (E)-hept-2-enal was superior to CA19-9 in the diagnosis of GBC, while others at most had a considerable diagnostic performance. To ensure the accuracy of the diagnostic model using limited data sets, a machine learning method was used to analyze the heterogeneous signal patterns of gallbladder diseases to obtain higher diagnostic accuracy. In the present study, four popular machine learning algorithms were used to construct the diagnostic model consisting of multiplexed indexes. Besides, the diagnostic accuracy of the VOC combination reached above 90%, which was superior to CA19-9. Support vector machines and linear discriminant analysis provided near 100% accuracy. Thus, we think bile VOCs panel is a suitable biomarker for GBC diagnosis.

## Conclusions

In the present study, our sample size was small, so it was difficult to avoid bias. Therefore, a large sample size and multicenter study should be carried out to further demonstrate the existing data. Meanwhile, it is necessary to further explore the relevant mechanism between the production of endogenous VOCs and the occurrence and development of GBC or BGD. This study provided an experimental basis for the application of VOC analysis in GBC and made it possible to be used in the early diagnosis of GBC, which had an extremely broad application prospect.

## Data Availability Statement

The original contributions presented in the study are included in the article/**Supplementary Material**. Further inquiries can be directed to the corresponding author.

## Ethics Statement

The studies involving human participants were reviewed and approved by the Ethics Committee of Qilu Hospital of Shandong University. The patients/participants provided their written informed consent to participate in this study.

## Author Contributions

Conceptualization, XZ, YiZ; methodology, XG, LZ; validation, XG, JG; investigation, YaZ, QL; data curation, JJ, JG; writing—original draft preparation, XZ; writing—review and editing, YaZ; visualization, LZ; supervision, YiZ; funding acquisition, XZ, JJ, YiZ. All authors have read and agreed to the published version of the manuscript.

## Funding

This research was funded by National Natural Science Foundation of China, 82172339 and 81972005, Natural Science Foundation of Shandong Province, ZR201910220159, Key Research and Development Program of Shandong Province, 2019GSF108064 and 2021CXGC011105, and Shandong Medical and Health Technology Development Project, 2018WS327.

## Conflict of Interest

Authors LZ and JG were employed by Hanon Advanced Technology Group Co.,Ltd,.

The remaining authors declare that the research was conducted in the absence of any commercial or financial relationships that could be construed as a potential conflict of interest.

## Publisher’s Note

All claims expressed in this article are solely those of the authors and do not necessarily represent those of their affiliated organizations, or those of the publisher, the editors and the reviewers. Any product that may be evaluated in this article, or claim that may be made by its manufacturer, is not guaranteed or endorsed by the publisher.
