# Supplementary figures and images for: A Panel of Bile Volatile Organic Compounds Servers as a Potential Diagnostic Biomarker for Gallbladder Cancer
Source: Front Oncol. 2022 Mar 30;12:858639. doi: 10.3389/fonc.2022.858639 (PMC9006947; doi:10.3389/fonc.2022.858639)

Supplementary Material


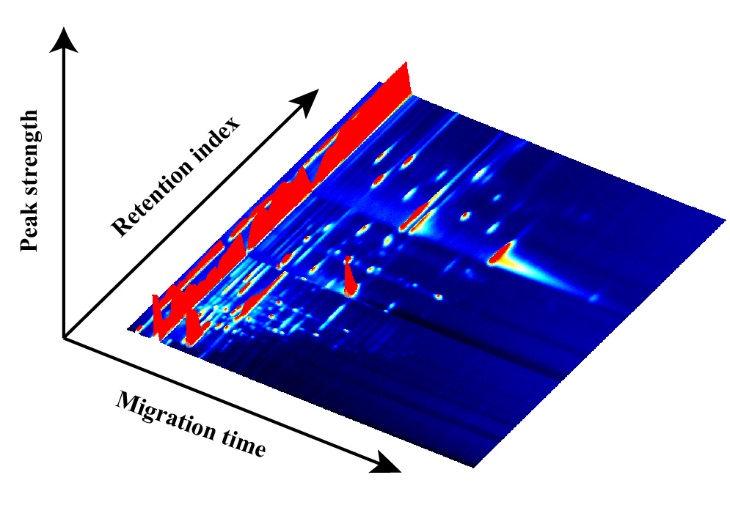


**Supplementary Figure 1.** 3D spectral map of VOC generated by IMS-GC.

Supplement: Supplementary file 1 [file DataSheet_1.docx]
