# Supplementary material for: A Panel of Bile Volatile Organic Compounds Servers as a Potential Diagnostic Biomarker for Gallbladder Cancer
Source: Front Oncol. 2022 Mar 30;12:858639. doi: 10.3389/fonc.2022.858639 (PMC9006947; doi:10.3389/fonc.2022.858639)
Supplement: Supplementary file 4 [file Table_3.docx]

**Table S3.** The levels of each volatile organic compound (μg/kg)

| VOCs | BGD | GBC | *P^#^* |
| --- | --- | --- | --- |
| Pentan-1-ol | 64.3(37.2-89.7) | 26.2(16.6-45.2) | <0.001 |
| (E)-2-octenal | 89.9(66.3-109.3) | 60.9(36.1-78.5) | <0.001 |
| (E)-hept-2-enal | 129.5(106.8-150.1) | 50.9(37.0-67.4) | <0.001 |
| Hexanal | 118.4(101.2-139.3) | 80.6(61.0-104.7) | <0.001 |
| (E)-2-hexenal | 88.6(41.9-121.0) | 33.9(29.4-48.3) | <0.001 |
| (E)-2-pentenal | 95.1(51.9-163.2) | 46.7(29.6-58.0) | <0.001 |
| Cyclohexanone | 131.6(119.8-332.9) | 571.1(374.9-596.7) | <0.001 |
| Acetophenone | 31.0(21.8-47.8) | 77.8(34.2-111.6) | <0.001 |
| Methyl benzoate | 8.8(6.1-20.0) | 14.5(10.4-45.3) | 0.006 |
| 1-octen-3-one | 40.9(19.5-61.6) | 24.2(13.6-29.8) | 0.008 |
| 2-ethyl-1-hexanol | 72.5(58.0-105.6) | 109(89.6-128.2) | 0.011 |
| Methyl acetate | 48.7(31.1-95.6) | 28.4(21.7-60.0) | 0.022 |
| Benzaldehyde | 29.1(20.9-50.5) | 38.7(28.5-56.7) | 0.120 |
| 2-heptanone | 62.4(40.0-81.9) | 61.2(40.9-76.0) | 0.667 |
| Nonanal | 34.2(21.4-57.0) | 23.12(18.57-30.7) | 0.057 |
| Methyl isobutyl ketone | 12.0(5.6-41.5) | 18.7(9.2-39.0) | 0.502 |
| 1-propene-3-methylthio | 8.8(6.2-13.0) | 9.1(5.1-17.5) | 0.790 |
| 2-Pentanone | 15.0(10.0-35.1) | 25.0(10.9-68.1) | 0.152 |
| Butyl acetate | 50.9(41.6-60.5) | 46.09(29.6-67.6) | 0.546 |

^#^Compared with Mann–Whitney U test, Data represents the median (interquartile range)
