# Supplementary material for: A Panel of Bile Volatile Organic Compounds Servers as a Potential Diagnostic Biomarker for Gallbladder Cancer
Source: Front Oncol. 2022 Mar 30;12:858639. doi: 10.3389/fonc.2022.858639 (PMC9006947; doi:10.3389/fonc.2022.858639)
Supplement: Supplementary file 5 [file Table_4.docx]

**Table S4.** The AUC, sensitivity and specificity of each machine learning model and CA19-9 for GBC diagnosis

| Model | AUC (95%CI) | Sensitivity (%) | Specificity (%) |
| --- | --- | --- | --- |
| DT | 0.917 (0.740-0.986) | 100 | 83.3 |
| KNN | 0.910 (0.730-0.984) | 87.5 | 94.4 |
| SVM | 0.972 (0.819-0.992) | 100 | 94.4 |
| LDA | 0.972 (0.819-0.992) | 100 | 94.4 |
| CA19-9 | 0.604 (0.395-0.788) | 87.5 | 33.3 |
